# The microtubule depolymerizing agent CYT997 effectively kills acute myeloid leukemia cells via activation of caspases and inhibition of PI3K/Akt/mTOR pathway proteins

**DOI:** 10.3892/etm.2013.1161

**Published:** 2013-06-14

**Authors:** XIAOHUI CHEN, CHUNMEI YANG, YANHUA XU, HUI ZHOU, HUI LIU, WENBIN QIAN

**Affiliations:** 1 Department of Hematology, The Affiliated Hospital of Hangzhou Normal University, Hangzhou, Zhejiang 310015;; 2 Institute of Hematology, The First Affiliated Hospital, College of Medicine, Zhejiang University, Hangzhou, Zhejiang 310003, P.R. China

**Keywords:** CYT997, apoptosis, acute myeloid leukemia

## Abstract

The orally active microtubule-depolymerizing agent CYT997 is potently cytotoxic to a variety of tumors *in vitro* and *in vivo*. However, the effects of this agent on acute myeloid leukemia (AML) cells and its mechanisms are unknown. The present study demonstrated that CYT997 effectively inhibited the growth of AML cells *in vitro*. Treatment of AML cells with CYT997 resulted in G2/M phase cell cycle arrest, and induced apoptosis through the activation of extrinsic and intrinsic apoptotic pathways. Furthermore, CYT997 induced cell death in CD123^+^ leukemia cells and significantly reduced leukemia colony formation. CYT997 was also demonstrated to exert dual effects on the expression of PI3K/Akt and mechanistic target of rampamycin (mTOR) signaling pathway proteins. Therefore, CTY997, used alone or in combination with chemotherapy, may represent a promising approach for the treatment of AML.

## Introduction

Acute myeloid leukemia (AML) is a heterogeneous malignancy characterized by the rapid growth of immature myeloid cells that undergo a differentiation block, resulting in an accumulation of leukemia cells in the bone marrow and the inhibition of normal hematopoiesis (1). Although intensive chemotherapy induces complete remission in the majority of patients with AML, a number of patients eventually relapse. The optimum strategy at the time of relapse or for patients with the refractory disease remains uncertain. Therefore, novel therapeutic approaches are required to improve the outcome of patients with AML. Microtubules are highly dynamic structures that are important in the maintenance of cell shape and organization of the mitotic spindle, which is necessary for mitosis (2). Microtubule-targeted agents (MTAs) are classified as destabilizing and stabilizing agents according to their binding site on tubulin or microtubules. MTAs exert a high cytotoxic efficiency, and also cause anti-angiogenic and vascular-disruptive effects (2,3). When utilized as anticancer agents, MTAs generally cause cell cycle arrest and apoptosis, as a consequence of caspase activation through the intrinsic apoptotic pathway. Similar effects have been observed in leukemia cells; certain MTAs, including PBOX-15 (4), CA4P (5) and PBOX-6 (6), displayed cytotoxic efficiency in various leukemia cell types through the inhibition of cellular proliferation. In addition, it has been demonstrated that CA4P, a novel tubulin-destabilizing agent, reduced the interaction of leukemia cells with neovessels by downregulating the expression of the adhesion molecule VCAM-1, thereby increasing leukemia cell death (5). Therefore, disrupting the microtubule function in leukemia cells is a potentially promising method for overcoming drug resistance in AML cells.

CYT997, a structurally novel orally active MTA, has been shown to inhibit tubulin polymerization and disrupt cellular microtubules (7). In addition, CYT997 has demonstrated potent cytotoxic activity against tumor cells, including hematopoietic malignancies *in vitro*; its effects are associated with the induction of apoptosis and cell cycle arrest at the G2/M phase (8–10). Notably, CYT997 also causes extensive ablation of the tumor vasculature, and thus inhibits the enlargement of tumors (10). In phase I clinical trials, the efficacy and safety of the drug has been investigated in patients with solid malignant tumors that were refractory to standard treatment (11,12). Eighteen patients (82%) that were treated with CYT997 for >2 cycles demonstrated a stable disease (11), indicating that CYT997 may be an important agent for the treatment of patients with refractory disease or may be used in combination with other anticancer therapies. In the present study, the *in vitro* effect of CYT997 on human AML cell lines was investigated. The cytotoxic mechanisms of CYT997 in leukemia cells were also investigated, with a particular focus on its effect on the cyclin-dependent kinase (cdc2) pathway, which regulates the entry of cells into mitosis, and the inhibition of PI3K/Akt/mTOR pathway proteins.

## Methods and materials

### Cell culture and reagents

Cell culture reagents, including RPMI-1640 and fetal bovine serum (FBS), were purchased from Gibco (Grand Island, NY, USA). z-IETD-FMK was obtained from BioVision (Palo Alto, CA, USA). Anti-CD123 antibody was purchased from BioLegend (San Diego, CA, USA) and CYT997 was obtained from Selleck (Houston, TX, USA). Methylcellulose and 3-(4,5-dimethylthiazol-2-yl)-2,5-diphenyltetrazolium bromide (MTT) were purchased from Sigma (St. Louis, MO, USA). All the antibodies used in the western blot analysis were purchased from Cell Signaling Technology, Inc., (Danvers, MA, USA). The human AML cell lines, K562, HL-60, KG-1, THP-1, Kasumi-1 and HEL, were obtained from the Institute of Hematology, Zhejiang University (Hangzhou, China). All leukemia cell lines were maintained in RPMI-1640 medium supplemented with 10% FBS at 37°C in a humidified atmosphere of 5% CO_2_.

### Cell viability assay

Cells were cultured at a density of 5×10^4^ cells/well in a 96-well plate and treated with CYT997 at concentrations of 12.5, 25, 50, 100 and 200 nM. Following 24 and 48 h of incubation, the medium was removed and fresh medium containing MTT was added to each well. This was followed by the addition of 200 *μ*l dimethyl sulfoxide (Amresco LLC, Solon, OH, USA), and 10 min of oscillation to dissolve the formazan crystals following 4 h of culture at 37°C. Subsequently, the mixture underwent centrifugation at 600 × g for 5 min, and the supernatant was discarded. The absorbance at 570 nm (A570) was measured using an enzyme-linked immunosorbent assay plate reader (Bio-Rad, Hercules, CA, USA).

### Annexin V binding assay

Cells were seeded in a 6-well plate and treated with CYT997 at concentrations of 0, 50, 100 and 200 nM. Following 24 h of treatment at 37°C, the cells were trypsinized and washed. Aliquots of the cells were resuspended in binding buffer and stained with 5 *μ*l Annexin V and 5 *μ*l propidium iodide (PI; Biouniquer, Nanjing, China) according to the manufacturer’s instructions. A fluorescence-activated cell-sorting (FACS) assay was performed immediately following the staining. To measure apoptosis in CD123^+^ cells, KG-1 cells were gated for CD123 expression, then the CD123^+^ cell subset was analyzed for positivity to Annexin V by flow cytometry following 24 or 48 h of treatment with CYT997 (100 nM).

### Cell cycle analysis

Following trypsinization, the cells were washed with phosphate-buffered saline (PBS) and subsequently fixed in 85% ethanol. Following fixation, the cells were washed with PBS/1% fetal calf serum (FCS), resuspended in PBS/1% FCS containing 10 *μ*g/ml PI and 250 *μ*g/ml RNase A, and incubated for 30 min at 37°C. Samples were tested using a FACSCalibur machine and CellQuest software (Becton-Dickinson, Mountain View, CA, USA).

### Leukemia colony-forming assay

The CYT997-treated cells were seeded in methylcellulose medium and incubated at 37°C in a humidified atmosphere with 5% CO_2_. Following 7 days of incubation, the number of leukemia colony-forming units (CFU-Ls) that contained >40 cells were scored manually under a light microscope (Olympus, Tokyo, Japan).

### Western blot analysis

Following treatment, cells were collected and lysed using 10 mM Tris, 1 mM ethylenediaminetetraacetic acid (EDTA), 10 mM KCl, and 0.3% Triton (pH 7.9). The concentration of the protein samples was measured by the Bradford method. The protein samples were separated by sodium dodecyl sulfate-polyacrylamide gel electrophoresis (SDS-PAGE) and then electroblotted onto Hybond-polyvinylidene fluoride (PVDF) membranes. The membranes were subjected to western blot analysis with primary antibodies to caspase-8, -9 and -3, poly ADP-ribose polymerase (PARP), Bid, phosphoinositide 3-kinase (PI3K) class III, Akt, phospho (p)-Akt, mechanistic target of rapamycin (mTOR), p-mTOR, p65, p-p65, cdc2, p-cdc2, cdc25c, p-cdc25c and β-actin.

### Statistical analysis

The experimental results are presented as the mean ± standard deviation. Statistical analysis was performed by the unpaired Student’s t-test. P<0.05 was considered to indicate a statistically significant difference.

## Results

### Effects of CYT997 on the viability of human AML cell lines

**Figure 1.**
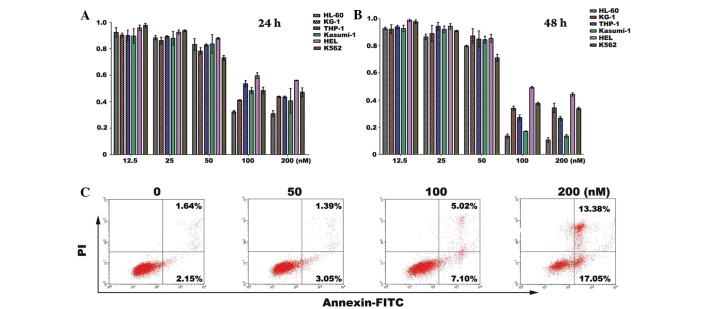
CYT997 inhibits cellular proliferation and induces apoptosis in acute myeloid leukemia (AML) cell lines. Cell viability was measured by a 3-(4,5-dimethylthiazol-2-yl)-2,5-diphenyltetrazolium bromide (MTT) assay in 96-well plates in the presence of increasing concentrations of CYT997 for (A) 24 h and (B) 48 h. The results represent the mean ± SD of four replicates. (C) HL-60 cells were treated with CYT997 (0, 50, 100, and 200 nM) for 24 h, resuspended in propidium iodide (PI)-containing hypotonic buffer, and then the percentage of apoptotic cells was analyzed by flow cytometry.

A panel of human AML cell lines and K562 cells was tested for sensitivity to CYT997 in a cell proliferation assay (Figs. 1A and B). Variability between the cell lines in sensitivity to CYT997 was indicated, regardless of the fact that cell growth inhibition occurred in a concentration- and time-dependent manner in all tested cell lines. Two cell lines, HL-60 and Kasumi-1, were particularly sensitive to the effects of CYT997. The IC_50_ values of CYT997 in HL-60, KG-1, THP-1, Kasumi-1 and HEL cell lines at 48 h were 60.75, 111.38, 96.06, 71.43 and 134.33 nM, respectively.

### Treatment with CYT997 resulted in the induction of apoptosis via activation of the caspase pathway

**Figure 2.**
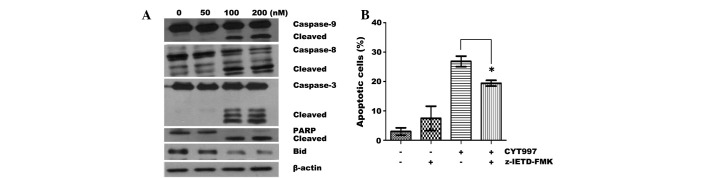
Activation of caspases by CYT997. (A) Immunoblotting analysis for caspase-9, -8 and -3 and poly ADP-ribose polymerase (PARP), was performed in lysates of HL-60 cells treated with CYT997 (0, 50, 100 or 200 nM) for 24 h. (B) Annexin V-propidium iodide (PI) staining was performed to quantify phosphatidylserine externalization in HL-60 cells treated with CYT997 (200 nM) or untreated for 24 h, in the presence or absence of the caspase-8 inhibitor. ^*^P<0.05.

Annexin V versus PI staining was used to assess the apoptotic status of HL-60 cells treated with increasing concentrations of CYT997. Leukemia cells treated with the drug exhibited a robust increase in apoptotic events, and the maximum number of apoptotic events were observed at 200 nM CYT997 (Fig. 1C). Western blot analysis was conducted to measure the activation of the caspase pathway. CYT997 triggered the concentration-dependent cleavage of caspase-8, -9 and -3, followed by cleavage of PARP (Fig. 2A). Moreover, treatment with CYT997 resulted in a reduction in the expression of the proapoptotic Bcl-2-related protein, Bid. This suggested that the Bid protein was truncated and thus may have resulted in the induction of the intrinsic pathway. As the effectiveness of MTAs is largely considered to be a consequence of caspase activation through the intrinsic apoptotic pathway (3), this study investigated the effect of the z-IETD-FMK caspase-8 inhibitor on CYT997-induced apoptosis. z-IETD-FMK (8 *μ*M) partially inhibited CYT997-induced apoptosis (Fig. 2B). These results suggest that treatment with CYT997 activates the cascades to the caspase-8 and -9 pathways in AML cells.

### CYT997 induced cell death in AML stem and progenitor cells

**Figure 3.**
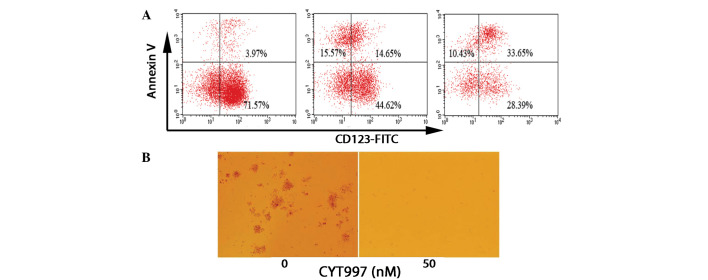
CYT997 induces apoptosis in the CD123^+^ acute myeloid leukemia (AML) cells and inhibits leukemia colony formation. (A) KG-1 cells were gated for CD123 expression, then the CD123^+^ population was analyzed for apoptosis by flow cytometry after 24 or 48 h of treatment with CYT997 (100 nM). (B) Following treatment with CYT997 at 50 nM, the colony formation of HL-60 leukemia cells was determined after 7 days of culture in methylcellulose medium. The photographic images were captured at ×40 magnification.

The interleukin-3 receptor α chain (CD123) has been demonstrated to be strongly expressed in AML stem cells, but not in human normal hematopoietic stem cells (13). The present study investigated whether CYT997 may induce apoptosis in leukemia progenitor cells. Following electronic gating on the CD123^+^ subpopulation, the cells were analyzed for positivity to Annexin V staining. KG-1 cells treated with CYT997 for 24 and 48 h demonstrated a time-dependent increase in the frequency of Annexin V^+^ and CD123^+^ cells, while the percentage of Annexin V^−^ and CD123^+^ cells significantly decreased (Fig. 3A). In the HL60-derived leukemia progenitor colony formation assays, CYT997 significantly reduced the colony formation ability (Fig. 3B). Collectively, the results suggest that CYT997 has cytotoxic activity against leukemia stem and progenitor cells.

### CYT997 inhibited PI3K/Akt/mTOR signaling

**Figure 4.**
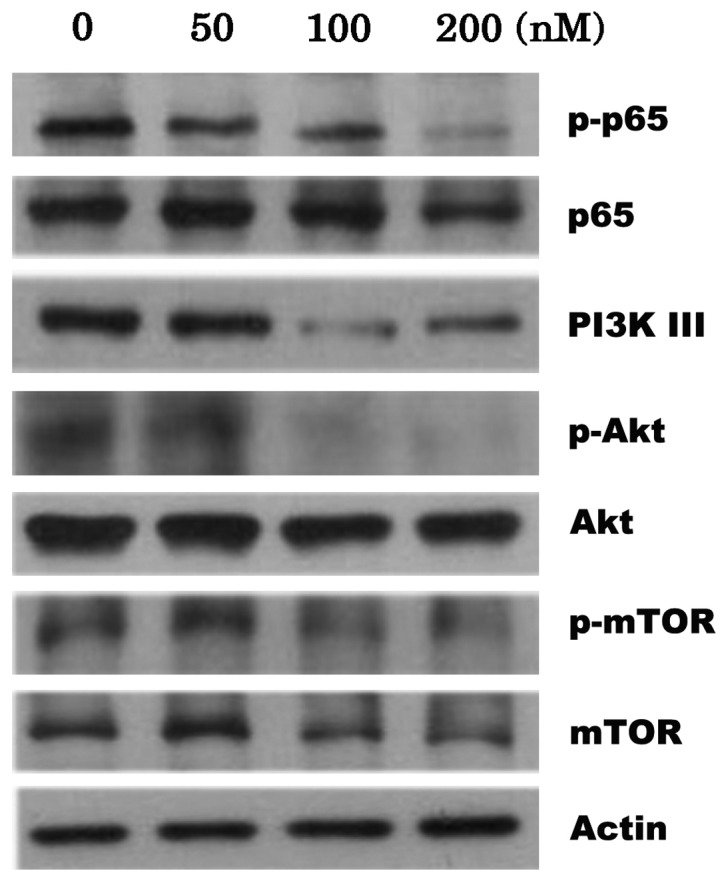
CYT997 affects the phosphorylation state of critical components of the phosphoinositide 3-kinase (PI3K)/Akt/mechanistic target of rapamycin (mTOR) signaling pathway in acute myeloid leukemia (AML) cells. HL-60 cells were treated with CYT997 at the indicated concentrations for 24 h. The cell extracts were prepared and then western blot analysis was performed using β-actin as a loading control.

The activation of the nuclear factor κB (NF-κB) pathway and the PI3K/AKT/mTOR axis has been shown to promote AML cell proliferation and contribute to drug resistance (14). Therefore, the present study investigated the mechanisms of CYT997, by studying possible alterations in the levels of proteins in the PI3K/Akt/mTOR pathway. The results indicated that CYT997 concentration-dependently reduced the phosphorylation of p65 and p-mTOR, accompanied by slight degradation of the proteins. Furthermore, treatment with CYT997 resulted in the downregulation of PI3K class III protein expression. In addition, the phosphorylation of Akt in the HL-60 cells was completely inhibited by treatment with 100 and 200 nM CYT997 (Fig. 4).

### CYT997 induced G2/M arrest in HL-60 cells via regulation of the expression of cell cycle regulatory proteins

**Figure 5.**
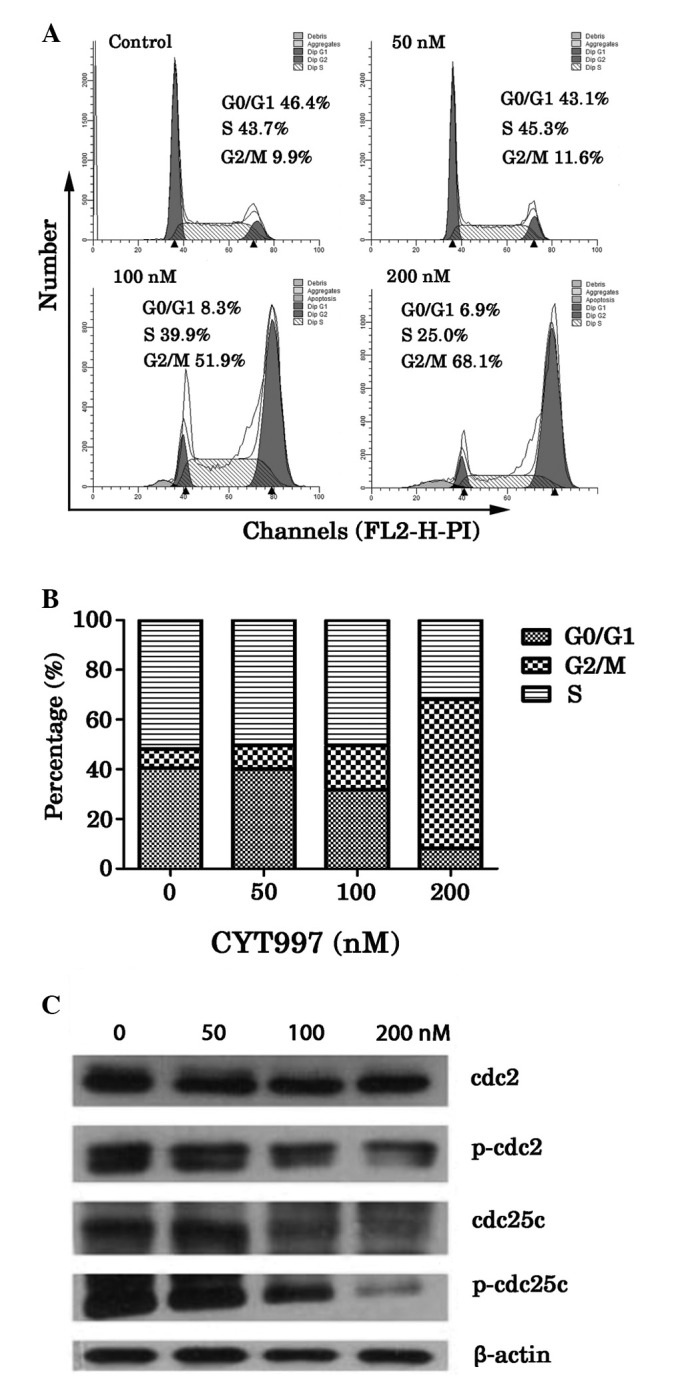
Effects of CYT997 on the cell cycle. (A and B) HL-60 cells were treated with CT997 (0, 50, 100 or 200 nM) for 24 h. The cell cycle was analyzed using propidium iodide (PI) staining followed by flow cytometry analysis. (C) HL-60 cells were cultured for 24 h in the presence of CYT997 at the indicated concentrations. Cell lysates were sepatated by sodium dodecyl sulfate-polyacrylamide gel electrophoresis (SDS-PAGE) and blotted to nitro-cellulose membranes that were incubated with antibodies to p-cdc2, cdc2, cdc25C, and p-cdc25C.

The present study determined the changes in the cell cycle following the treatment of HL-60 cells with CYT997 for 24 h. The percentage of cells in the G2/M phase increased following treatment with CYT997 in a concentration-dependent manner; the percentage increased from 9.9% in the absence of CT997 to 11.6, 51.9 and 68.1% in the presence of 50, 100 and 200 nM CT997, respectively (Fig. 5). These results suggest that CYT997 induced G2/M arrest in the AML cells, which is consistent with previous results obtained using other MTAs including PBOX-6, paclitaxel and vincristine (15). In the present study, the western blot analysis identified that CYT997 downregulated the phosphorylated form of cdc2, while the expression of cdc2 did not change. CYT997 also mediated inhibition of the cell cycle protein involved in the regulation of G2 to M phase transition, namely cdc25C (Fig. 5C).

## Discussion

Conventional antimicrotubule compounds have been clinically used to treat patients with cancer; however, their neurotoxicity and the emergence of acquired resistance limit the success of therapy (16,17). There has been significant focus on the discovery and development of novel, small, molecular microtubule-targeted agents with a higher antitumor efficacy and lower toxicity (8). It has been demonstrated that PBOX compounds, a novel series of microtubule-depolymerizing agents, are capable of inducing apoptosis in drug-resistant HL60 cells expressing P-glycoprotein or breast cancer resistance protein (BCRP) (15). Furthermore, CA4P, a microtubule-destabilizing agent, not only inhibits leukemia cell proliferation, but also causes a reduction in the interaction of leukemia cells with neovessels, thereby augmenting leukemia cell death *in vivo* (5). The present study demonstrated that CYT997 induced the apoptosis and inhibited the growth of AML cell lines including HL-60, KG-1, THP-1, Kasumi-1 and HEL, with IC_50_ values of 60.75–134.33 nM at 48 h. These results are comparable with those from a previous study where the IC_50_ values for various solid tumor cell lines at 72 h were 9–101 nM (8). Notably, the present study showed that treatment with 100 nM CYT997 for 24 and 48 h resulted in marked levels of apoptosis in CD123^+^ KG-1 cells. Similar to these results, the colony formation assay indicated a complete cessation of leukemia colony formation at 50 nM in the HL-60 cells. Therefore, the results demonstrated that CYT997 effectively induced cytotoxicity against AML cells, and leukemia stem and progenitor cells.

CYT997-induced apoptosis in the AML cell lines was associated with the significant activation of caspase-3, -8 and -9. Moreover, pretreatment with Z-IETD-FMK, a caspase-8 inhibitor, partially inhibited the CYT997-induced apoptosis. These results indicate that CYT997 induced apoptosis through activating the caspase pathway, including the extrinsic and intrinsic apoptotic pathways. The activation of Bid represents an important mechanism accounting for cross-talk between the extrinsic and intrinsic apoptotic pathways. In the current study, CYT997 significantly downregulated Bid expression, suggesting that cleavage of Bid was induced by the agent. Furthermore, it has been demonstrated that the activation of the PI3K/Akt/mTOR axis is a common feature in patients with AML, and inhibition of mTOR blocks the phosphorylation of this kinase and results in cell death in leukemia progenitor cells (14,18–20). The present study demonstrated that CYT997 inhibited PI3K class III protein expression and decreased the levels of p-65. CYT997 also induced the downregulation of the expression levels of p-mTOR and mTOR, and completely eliminated p-Akt. Previous studies have identified that inhibition of the PI3K/Akt pathway by a specific inhibitor failed to induce the apoptosis of AML cells, but co-inhibition of the PI3K and mTOR pathways significantly induced apoptosis (21,22). Therefore CYT997, which kills AML cells by activation of the caspase pathway and dual inhibition of the PI3K/Akt and mTOR pathways, may be useful for overcoming drug resistance.

Due to their involvement in mitosis, disruption of the function of microtubules results in the arrest of the cell cycle at G2 phase, in which the cyclin-dependent kinase (CDK1)/cyclin B complex is important for progression from the G2 to the M phase (23). The activation of the CDK1/cyclin B complex is maintained by cdc25 phosphatase. Dephosphorylation of CDK1 is catalyzed by cdc25C (24). In the present study, phosphorylation of CDK1, also known as cdc2, was remarkably inhibited by CYT997. The results demonstrated that the levels of cdc25C and p-cdc25C were reduced in cells treated with CYT997. Therefore, CYT997 treatment induced a typical G2/M cell cycle arrest in the AML cells by regulating CDK1 phosphorylation and cdc25C expression.

In conclusion, the present study demonstrated that CYT997 inhibited the proliferation of AML cells and induced apoptosis through the activation of the extrinsic and intrinsic apoptotic pathways. CYT997 induced cell death in CD123^+^ leukemia cells and reduced leukemia colony formation. Furthermore, the drug exerted dual effects on the expression of PI3K/Akt and mTOR signaling pathway proteins. These results suggested that CTY997, used alone or in combination with chemotherapy, may represent a promising approach for the treatment of AML.
